# Triptolide Upregulates Myocardial Forkhead Helix Transcription Factor p3 Expression and Attenuates Cardiac Hypertrophy

**DOI:** 10.3389/fphar.2016.00471

**Published:** 2016-11-30

**Authors:** Yuan-Yuan Ding, Jing-Mei Li, Feng-Jie Guo, Ya Liu, Yang-Fei Tong, Xi-Chun Pan, Xiao-Lan Lu, Wen Ye, Xiao-Hong Chen, Hai-Gang Zhang

**Affiliations:** ^1^Department of Pharmacology, College of Pharmacy, Third Military Medical UniversityChongqing, China; ^2^The People’s Liberation Army No. 309 HospitalBeijing, China; ^3^Institute of Materia Medica and Department of Pharmaceutics, College of Pharmacy, Third Military Medical UniversityChongqing, China; ^4^Department of Pharmacy, Chongqing Traditional Medicine HospitalChongqing, China; ^5^Department of Clinical Laboratory, First Affiliated Hospital of North Sichuan Medical CollegeNanchong, China

**Keywords:** cardiac hypertrophy, cardiac fibrosis, forkhead box transcription factor p3, triptolide, cardiac injury

## Abstract

The forkhead/winged helix transcription factor (Fox) p3 can regulate the expression of various genes, and it has been reported that the transfer of Foxp3-positive T cells could ameliorate cardiac hypertrophy and fibrosis. Triptolide (TP) can elevate the expression of Foxp3, but its effects on cardiac hypertrophy remain unclear. In the present study, neonatal rat ventricular myocytes (NRVM) were isolated and stimulated with angiotensin II (1 μmol/L) to induce hypertrophic response. The expression of Foxp3 in NRVM was observed by using immunofluorescence assay. Fifty mice were randomly divided into five groups and received vehicle (control), isoproterenol (Iso, 5 mg/kg, s.c.), one of three doses of TP (10, 30, or 90 μg/kg, i.p.) for 14 days, respectively. The pathological morphology changes were observed after Hematoxylin and eosin, lectin and Masson’s trichrome staining. The levels of serum brain natriuretic peptide (BNP) and troponin I were determined by enzyme-linked immunosorbent assay and chemiluminescence, respectively. The mRNA and protein expressions of α- myosin heavy chain (MHC), β-MHC and Foxp3 were determined using real-time PCR and immunohistochemistry, respectively. It was shown that TP (1, 3, 10 μg/L) treatment significantly decreased cell size, mRNA and protein expression of β-MHC, and upregulated Foxp3 expression in NRVM. TP also decreased heart weight index, left ventricular weight index and, improved myocardial injury and fibrosis; and decreased the cross-scetional area of the myocardium, serum cardiac troponin and BNP. Additionally, TP markedly reduced the mRNA and protein expression of myocardial β-MHC and elevated the mRNA and protein expression of α-MHC and Foxp3 in a dose-dependent manner. In conclusion, TP can effectively ameliorate myocardial damage and inhibit cardiac hypertrophy, which is at least partly related to the elevation of Foxp3 expression in cardiomyocytes.

## Introduction

Cardiac hypertrophy is a pathological feature of various cardiac diseases, including hypertension, cardiomyopathy, valvular dysfunction, myocardial infarction, etc., and also the strongest predictor for the development of heart failure, arrhythmia, and sudden death (van Berlo et al., 2013; Frieler and Mortensen, 2015). It has been identified as a significant independent risk factor for cardiac diseases and suggested to be the primary therapeutic goal for hypertension and chronic heart failure (Chemaly et al., 2013; Verschuren et al., 2014; Ikeda et al., 2015). In order to maintain cardiac output, some compensatory mechanisms are activated, such as the Frank-Starling response, neuroendocrine activation, and inflammatory reaction (van Berlo et al., 2013; Kessler et al., 2014). Inflammation and the activation of immune system are involved in orchestrating this complex pathological response. It has been shown that the functions of regulatory T (Treg) cells are decreased in patients with chronic heart failure and cardiac hypertrophy (McKinsey, 2012), which implies that Treg cells are involved in the pathophysiological process of cardiac hypertrophy.

The forkhead/winged helix transcription factor (Fox) p3 is a key transcription factor that largely controls the phenotype and function of Treg cells (Hori et al., 2003). And Treg cells also need continuous high expression of Foxp3 to maintain immune homeostasis (Sakaguchi et al., 2008; Klein et al., 2013). Foxp3 can depress the activation of immune cells and their migration to myocardium (Matsumoto et al., 2011), reduce the cardiac interstitial inflammatory cell infiltration and the secretion of cytokines, and then ameliorate cardiac hypertrophy and myocardial damage induced by angiotensin II (AngII) or high blood pressure. It has been reported that adoptive transfer of Treg cells into AngII–infused hypertensive mice could improve cardiac hypertrophy and ameliorate cardiac fibrosis despite sustained hypertension (Kvakan et al., 2009; Kasal et al., 2012).

Although Foxp3 has been considered as a characteristic marker of Treg cells, it is found to be expressed in a variety of cells, such as other immune cells, hematopoietic cells, epithelial cells and tumor cells from multiple sources (Katoh et al., 2010; Ladoire et al., 2011; Kasal et al., 2012). Regarding its role as a transcription regulator, its regulatory mechanism in the process of cardiac hypertrophy is still unclear.

Triptolide (TP) is an epoxy-2-terpenoid ester compound extracted from the Chinese traditional herb Leigongteng (*Tripterygium wilfordii* Hook. f.), which has been used for the treatment of rheumatoid arthritis, systemic lupus erythematosus, discoid lupus erythematosus, psoriasis, asthma and cancers (Liu, 2011; Fan et al., 2016; Ziaei and Halaby, 2016). It has been suggested that TP treatment could enhance the expression of Foxp3 in CD4^+^ cells (Zhang et al., 2009; Zheng et al., 2013) and attenuate the development of pulmonary arterial neointimal formation (Faul et al., 2000) and the proliferation of fibroblasts in the heart and airway (Leonard et al., 2002; Zhang et al., 2013). However, the effects of TP on cardiac hypertrophy and its mechanism are at present poorly understood. Therefore, the aims of the present study were to explore the regulating effect of TP on myocardial hypertrophy and its relation to Foxp3 *in vitro* and *in vivo*.

## Materials and Methods

### Animals

Fifty healthy male Kunming mice weighing 20–28 g were randomly divided into 5 groups (*n* = 10 in each group), i.e., normal control, myocardial hypertrophy model group and TP (10, 30, 90 μg/kg) treated groups. Mice in the model group (isoproterenol treated group, Iso) and TP groups were injected subcutaneously with (±)isoproterenol hydrochloride (Sigma, St. Louis, MO, USA) 5 mg/kg once daily for 14 days to induce cardiac hypertrophy according to the method described by Ma and other researchers (Ma et al., 2011; Song et al., 2013). Animals in the TP groups were intraperitoneally injected with TP (purity 99.69%; Beijing Medicass Biotechnol, Beijing, China) at doses of 10, 30, or 90 μg/kg daily, respectively, for 14 days. Those in the control group were injected with an equal volume of normal saline simultaneously. The mice in all groups were weighed every 3 days and the doses were adjusted accordingly. All animals were housed under conditions of controlled temperature (20–25°C) and humidity (60–65%), and a 12-h light-dark cycle and were fed with standard food and water *ad libitum*. This investigation conformed to *The Guide for the Care and Use of Laboratory Animals* published by the US National Institutes of Health (NIH Publication No. 85-23, revised 1996) and was approved by the Ethical Committee for Animal Experimentation of the Third Military Medical University. Humane end points were set according to the *OECD Guidance Document on the Recognition, Assessment, and Use of Clinical Signs as Humane End points for Experimental Animals Used in Safety Evaluation ^1^* .

### Sampling

At the end of treatments, all animals were weighted and anesthetized with pentobarbital sodium (50 mg/kg, i.p.). Blood was sampled from the abdominal aorta and allowed to coagulate at 37°C for 2 h. After centrifugation at 4000 rpm for 10 min, the suspension were collected and stored at -20°C. The mice were decapitated and the hearts were excised and placed in a dish with normal saline, then blotted on filter paper and weighed to calculate the ratio of heart weight to body weight (HW/BW) and the ratio of left ventricular weight to body weight (LVW/BW). Tibial length (TL) was measured to calculate the ratio of heart weight or left ventricular weight to tibial length (HW/TL, LVW/TL). The mid-ventricle was fixed with a formalin neutral buffer solution and embedded in paraffin. The apex of the ventricle was stored in liquid nitrogen for future use.

### Cell Culture

Neonatal rat ventricular myocytes (NRVM) from 1 to 2 days old Sprague-Dawley rats were isolated and cultured as described previously (Yang et al., 2013; Lu et al., 2015). After being cultured in serum-free DMEM for 24 h, the cells were incubated for 24 h in a non-serum medium containing 1 μmol/L AngII. Different concentrations of TP (1, 3, or 10 μg/L) were added simultaneously. The cell size was determined with rhodamine-labeled phalloidin staining and analyzed with ImageJ software (NIH Image, National Institutes of Health, Bethesda, MD, USA^2^).

### Immunofluorescence Staining

Neonatal rat ventricular myocytes were isolated and cultured as described above. Myocytes cultured on glass coverslips were fixed with 4% (w/v) paraformaldehyde for 30 min at room temperature. NRVMs were subjected to immunofluorescence with a primary α-actinin polyclonal rabbit antibody and primary Foxp3 mouse monoclonal antibody (1:100, Santa Cruz Biotechnology, Santa Cruz, CA, USA), and a secondary anti-rabbit antibody conjugated to Alexa Fluor 555 and secondary anti-mouse antibody conjugated to fluor 488, respectively. Cells were co-stained with DAPI to visualize their nuclei. A negative control was carried out by replacing the primary antibody with isotype IgG. The expression of Foxp3 in NRVMs was observed by laser scanning confocal microscopy (LSM 780 AxioObserver, Carl Zeiss Microscopy, München, Germany) and analyzed by ZEN imaging software (2012, blue edition, Carl Zeiss).

### Western Blot Assay

The β-MHC expression of NRVM was determined with Western blotting method. Total cellular homogenates were prepared, and 30 μg of the denatured proteins were loaded and separated using SDS-PAGE. The proteins were transferred onto a PVDF membrane (Millipore, Bedford, MA, USA) and incubated with a primary β-MHC antibody (1:1000; Santa Cruz Biotechnology, Santa Cruz, CA, USA) at 4°C overnight, followed by horseradish peroxidase (HRP)-conjugated secondary antibody. Chemiluminescence was detected with an ECL detection kit (Millipore, Bedford, MA, USA).

### Morphometric Analysis of Myocardial Tissue

Left ventricle tissue was fixed in 10% formalin for 48 h and was embedded in paraffin. Sections (5 μm) were stained with hematoxylin and eosin (HE), fluorescein isothiocyanate (FITC)-labeled lectin wheat germ agglutinin (Sigma, St. Louis, MO, USA), as well as Masson’s trichrome (Chen et al., 2011). Cross-sectional areas (CSA) were measured using the Image Pro Plus 5.1 image analysis program (Media Cybernetics, Silver Spring, MD, USA). Fibrosis percentage was calculated as the ratio of fibrotic area to total LV area (Pereira et al., 2014; Matsuo et al., 2015).

### Immunohistochemistry

The paraffin sections were manufactured as described previously. α-MHC, β-MHC and Foxp3 monoclonal antibodies (Santa Cruz Biotechnology, Santa Cruz, CA, USA) were used at a dilution of 1:200 according to the manufacturer’s instruction. To visualize this reaction after incubation with a secondary antibody at room temperature, the slides were incubated with 3,3 *N*-diaminobenzidine tetrahydrochloride (DAB). The reaction was stopped by immersing the slides in distilled water, and then the sections were counterstained with haematoxylin, mounted and examined. The OD values were calculated by Image Pro-Plus5.1 (Media Cybernetics, Silver Spring, MD, USA).

### Serum Troponin I Assay

The levels of serum troponin I (cTnI) were measured with the Abbott automatic chemiluminescence immunoassay analyzer (Architect i2000SR; Abbott Diagnostics, Abbott Park, IL, USA). The detection routine was referred to FLEX TM technology with cTnI reagent.

### Serum Brain Natriuretic Peptide Assay

The levels of serum brain natriuretic peptide (BNP) were quantified by enzyme-linked immunosorbent assay (ELISA) kits and performed according to the manufacturer’s instructions (R&D systems, Minneapolis, MN, USA) in accordance with the Double-antibody Sandwich ELISA instructions. The BNP values were calculated by standard curve method.

### Real-Time Reverse Transcription PCR

Total RNA was extracted from NRVM and LV tissue using TRIzol reagent (Invitrogen, Carlsbad, CA, USA) according to the manufacturer’s instructions. Only highly pure RNAs (OD_260_/OD_280_ in the range of 1.80–2.10) were used for downstream assays. RNA was reverse transcribed with the PrimeScript RT reagent kit (TaKaRa, Japan). Real-time PCR for α-MHC, β-MHC and Foxp3 mRNA was performed using the following primers designed by Premier 5.0 (Premier Biosoft International, Palo Alto, CA, USA) for α-MHC (forward: 5′-GCCGAGTCCCAGGTCAACA-3′, reverse: 5′-TATTGGCCACAGCGAGGGTCT-3′), β-MHC (forward: 5′-GGCAAGACGGTG ACTGTGAAGG-3′, reverse: 5′-GGTTGACGGTGACGCAGAAGAG-3′), ANP (forward: 5′-GAGGAGAAGATGCCGGTAG-3′; reverse: 5′-CTAGAGAGGGAGCT AAGTG-3′), Foxp3 (forward: 5′-ACTGGGCTTCTGGGTATGTC-3′; reverse: 5′-TAGCTTGCG GCTCCTAATGC-3′), and β-actin (forward: 5′-GTCCCTCACCC TCCCAAAAGC-3′; reverse: 5′-CACAGAAGCAATGCTGTCACCT-3′). The reaction was performed in a total volume of 25 μl with QPK-201 SYBR Green PCR Master Mix (Takara, Shiga, Japan) using the following conditions: after an initial 5 min at 94°C, the samples were submitted to 38 cycles comprising 30 s at 94°C for denaturation, 30 s at 62°C for annealing, and 45 s at 72°C for elongation. Finally, 10 min at 72°C for ending this reaction. The Ct (cycle threshold) values were normalized to both the β-actin expression level and the normal controls, and the relative quantification was calculated using the 2^-ΔΔCt^ method (Livak and Schmittgen, 2001; Zwadlo et al., 2015).

### Statistical Analysis

The results are expressed as the mean ± standard error of mean (SEM). The data were analyzed by one-way analysis of variance (ANOVA) with least significant difference (LSD) *post hoc* analyses (SPSS, Chicago, IL, USA) and GraphPad Prism version 5.01 (GraphPad Software, La Jolla, CA, USA). The survival rate was compared with χ^2^ test. The value of *P* less than 0.05 was considered statistically significant.

## Results

### Hypertrophic Response of NRVM

**FIGURE 1:**
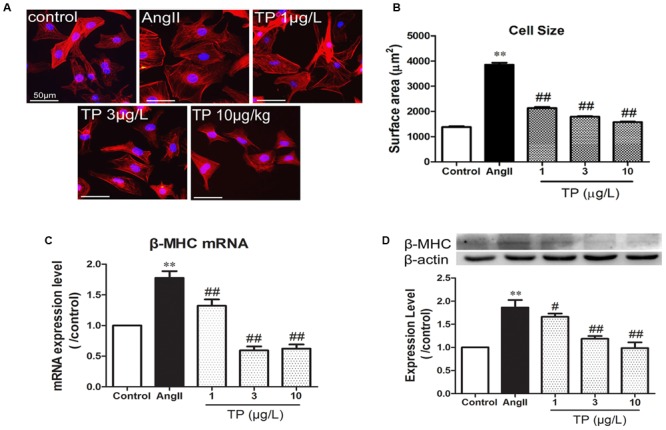
**Triptolide attenuated the hypertrophic response of neonatal rat ventricular myocytes.** Hypertrophic response of neonatal rat ventricular myocytes (NRVM) was induced by angiotensin II (Ang II). **(A)** NRVM treated with Ang II (1 μmol/L) and triptolide (TP) for 24 h, stained with rhodamine-phalloidin (bar = 50 μm); **(B)** cell size (*n* = 50 cells in each group); **(C)** β-MHC mRNA expression determined using Real-time PCR (*n* = 4); **(D)** β-MHC expression level determined using Western blotting (*n* = 4). The data are presented as mean ± SEM, ^∗∗^*p* < 0.01 compared with the control group; ^#^*p* < 0.05, ^##^*p* < 0.01 compared with the Ang II-treated group (one-way ANOVA).

After stimulation with AngII for 24 h, the surface area of primary cardiomyocytes significantly increased by 1.83-folds compared to the control (AngII vs. control: 3849 ± 81 vs. 1379 ± 31 μm^2^; *p* < 0.01). The three dose of TP (1, 3 or 10 μg/L) treatment reduced cardiomyocyte size significantly (**Figures 1A,B**). Moreover, the mRNA and protein expression of β-MHC, which is a marker of cardiac hypertrophy and is induced by disease-related hypertrophic stimuli in rat ventricular myocytes, increased dramatically after AngII stimulation (**Figures 1C,D**). Simultaneously, TP decreased the expressions of β-MHC and its mRNA significantly by comparison to those in AngII group, respectively.

### Expression of Foxp3 in NRVM

**FIGURE 2:**
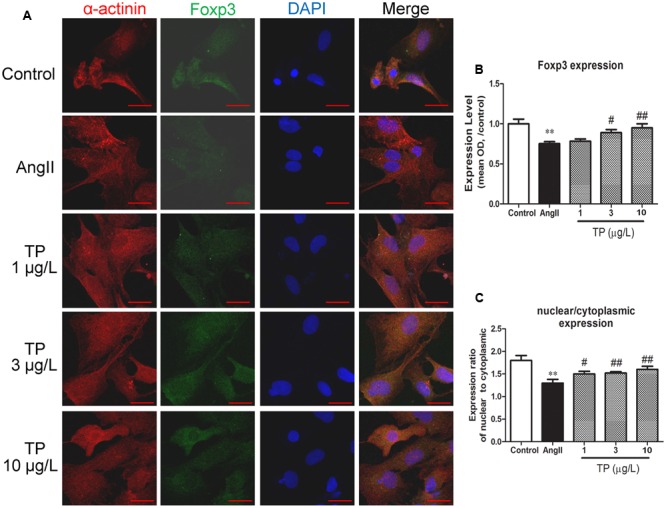
**Expression of transcription factor Foxp3 in neonatal rat ventricular myocytes (NRVM).** Hypertrophic response of NRVM was induced by angiotensin II (Ang II). **(A)** Expression of Foxp3 (green) was detected using double-label immunofluorescence staining with α-actinin (red). Cells were co-stained with DAPI to visualize their nuclei (blue). Images from the same field were merged. (bar = 20 μm); **(B)** Expression intensity level of Foxp3; **(C)** Ratio of Foxp3 expression in nucleus to that in cytoplasm. The data are presented as mean ± SEM, ^∗∗^*p* < 0.01 compared with the control group;^#^*p* < 0.05, ^##^*p* < 0.01 compared with the Ang II-treated group (one-way ANOVA).

Double-immunofluorescence staining was used to colocalize Foxp3 (green) with cardiomyocytes (anti-α-actinin, red). Foxp3 was expressed in both the cytoplasm and nuclei (**Figure 2A**), and the expression intensity in the nuclei was significantly higher than that in the cytoplasm in all groups (*p* < 0.01). Compared with the control group, the expression level of Foxp3 in myocytes treated with AngII decreased markedly. TP (3 and 10 μg/L) could significantly elevate Foxp3 expression by comparison to that in the AngII-treated group (**Figure 2B**). Furthermore, the ratio of nuclear to cytoplasmic expression of Foxp3 decreased after AngII treatment (*p* < 0.01) and increased in TP treated groups (*p* < 0.05 or 0.01) (**Figure 2C**).

### Survival of Mice

During the whole experimental period, one mouse in the control group, two mice in the Iso group and one in the 10 μg/kg TP group were sacrificed before the humane endpoints. There were no significant differences in mortality between all the groups.

### Myocardial Hypertrophy

**FIGURE 3:**
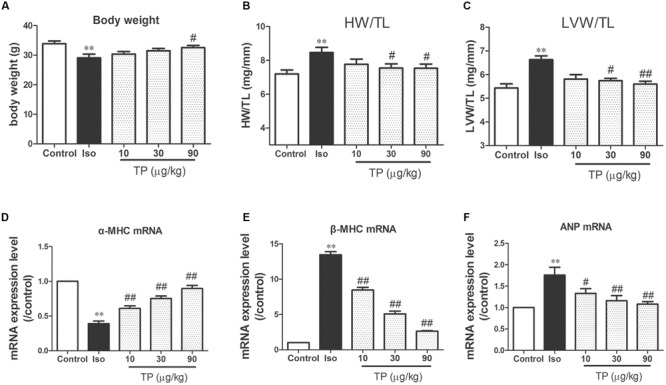
**Triptolide attenuated cardiac hypertrophy in mice.** Cardiac hypertrophy was induced by isoproterenol (Iso, 5 mg/kg, s.c., for 14 days) in mice (*n* = 8–10 in each group). **(A)** Body weight; **(B)** heart weight (HW) index to tibia length (TL); **(C)** left ventricular weight (LVW) indexes to TL; **(D)** α-myosin heavy chain (MHC) mRNA expression level; **(E)** β-MHC mRNA expression level; **(F)** atrial natriuretic peptide (ANP) mRNA expression level. These mRNA expression levels were measured with Real-time PCR method and normalized to β-actin and control group, respectively. The data are presented as mean ± SEM, ^∗∗^*p* < 0.01 compared with the control group; ^#^*p* < 0.05, ^##^*p* < 0.01 compared with the Iso-treated group (one-way ANOVA).

The heart and LV weight indexes were measured to evaluate the cardiac hypertrophic response. The weight of the heart and LV in the Iso group were increased 15.6% (*p* < 0.05) and 19.6% (*p* < 0.05) respectively compared to those in the control group. After treatment with TP 30 and 90 μg/kg, all the heart indexes, including HW/BW, LVW/BW, HW/TL, and LVW/TL, were decreased markedly compared with those in the Iso group (*p* < 0.05 or 0.01). In the 30 and 90 μg/kg group, TP reduced the ratio of LV to TL by 13.4 and 15.6%, respectively (**Figures 3A–C**).

β-MHC, ANP and the ratio of β-MHC to α-MHC are considered as the molecular markers of myocardial hypertrophy (Wang et al., 2002, 2005). mRNA expression level of these three genes were determined using real-time PCR. Iso treatment markedly decreased α-MHC mRNA expression level, and elevated mRNA expression level of β-MHC and ANP, as well as the ratio of β-MHC to α-MHC mRNA. TP significantly downregulated the expression of β-MHC and ANP mRNA compared with those in Iso group (**Figures 3D–F**).

### Histological Findings

**FIGURE 4:**
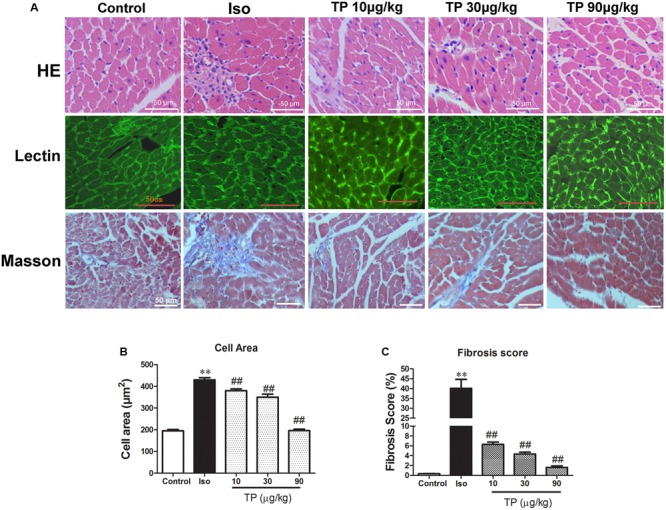
**Myocardial histological changes after triptolide treatment.** Cardiac hypertrophy was induced by isoproterenol (Iso, 5 mg/kg, s.c., for 14 days) in mice (*n* = 8–10 in each group). The mice were treated with normal saline, Iso, and triptolide (10, 30, 90 μg/kg), respectively, for 14 days. **(A)** hematoxylin and eosin (HE) staining (bar = 50 μm), Wheat germ agglutinin lectin staining (bar = 50 μm) and Masson’s trichome staining (bar = 50 μm); **(B)** Cross-sectional area; **(C)** Fibrosis score. The data are presented as mean ± SEM, ^∗∗^*p* < 0.01 compared with the control group; ^##^*p* < 0.01 compared with the Iso-treated group (one-way ANOVA).

Under a light microscope, we found hypertrophic cardiomyocytes, myocardial fiber disruption, focal necrosis and inflammatory cell infiltration in the Iso group (**Figure 4A**, HE staining). Iso treatment resulted in a significant increase in the cross-sectional area (CSA) of cardiomyocytes and LV interstitial fibrosis compared with the control group. TP (10, 30, or 90 μg/kg) treatment significantly decreased myocardial tissue damage and decreased CSA and fibrosis score compared with Iso group (**Figures 4A–C**).

### Myocardial Expression of α-MHC and β-MHC

**FIGURE 5:**
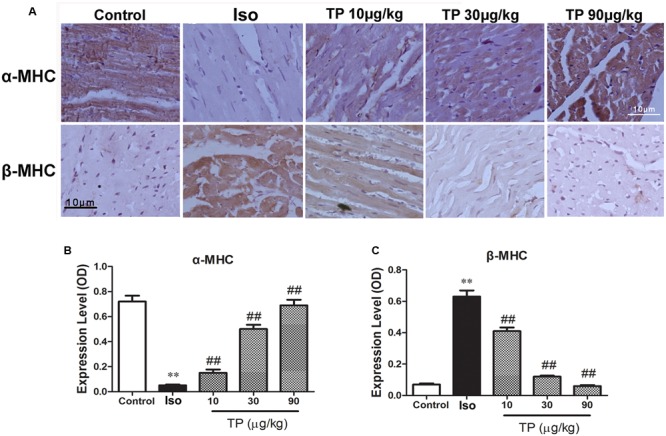
**The effects of triptolide on the myocardial expression of myosin heavy chains (MHC).** Cardiac hypertrophy was induced by isoproterenol (Iso, 5 mg/kg, s.c., for 14 days) in mice (*n* = 8–10 in each group). The mice were treated with normal saline, Iso, and triptolide (10, 30, 90 μg/kg), respectively, for 14 days. **(A)** The expression of α- and β-MHC were determined with immunohistochemistry (bar = 10 μm); **(B)** Expression of α-MHC; **(C)** Expression of β-MHC. The data are presented as mean ± SEM, ^∗∗^*p* < 0.01 compared with the control group; ^##^*p* < 0.01 compared with the Iso-treated group (one-way ANOVA).

The protein expressions of α-MHC and β-MHC were determined using immunohistochemistry technique. The result demonstrated that in the control group α-MHC was expressed at a high level, but β-MHC was expressed at a hardly detectable level. Compared with control, the expression of α-MHC was down-regulated and β-MHC was increased in the Iso group significantly (*p* < 0.01). TP treatment could elevate α-MHC expression but decrease β-MHC substantially. The ratio of α-MHC to β-MHC, an important index which contributes to contractile function, decreased in the Iso group and could be augmented by TP treatment strikingly (**Figures 5A–C**).

### Serum Concentration of cTnI and BNP

**FIGURE 6:**
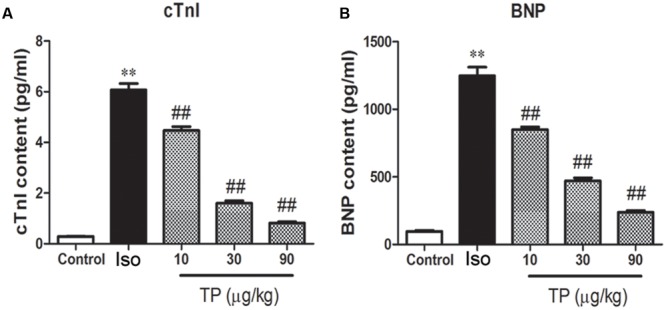
**The effects of triptolide on serum cardiac troponin-I (cTnI) (A)** and brain natriuretic peptide (BNP) **(B)** in mice. Cardiac hypertrophy was induced by isoproterenol (Iso, 5 mg/kg, s.c., for 14 days) in mice (*n* = 8–10 in each group). The mice were treated with normal saline, Iso, and triptolide (10, 30, 90 μg/kg), respectively, for 14 days. The data are presented as mean ± SEM, ^∗∗^*p* < 0.01 compared with the control group; ^##^*p* < 0.01 compared with the Iso-treated group (one-way ANOVA).

To evaluate myocardial injury, the serum concentrations of cTnI and BNP were determined with chemiluminescence immunoassay and ELISA, and they increased by 5.78-fold and 11.87-fold, respectively, compared to the control group (**Figure 6**). TP decreased the concentrations of cTnI and BNP markedly in a dose-dependent manner. cTnI in the TP (10, 30, 90 μg/kg) groups decreased 26.4, 73.6, and 86.5%, and BNP decreased 31.9, 62.3, and 80.8%, respectively, compared with the Iso groups.

### Myocardial Expression of Foxp3

**FIGURE 7:**
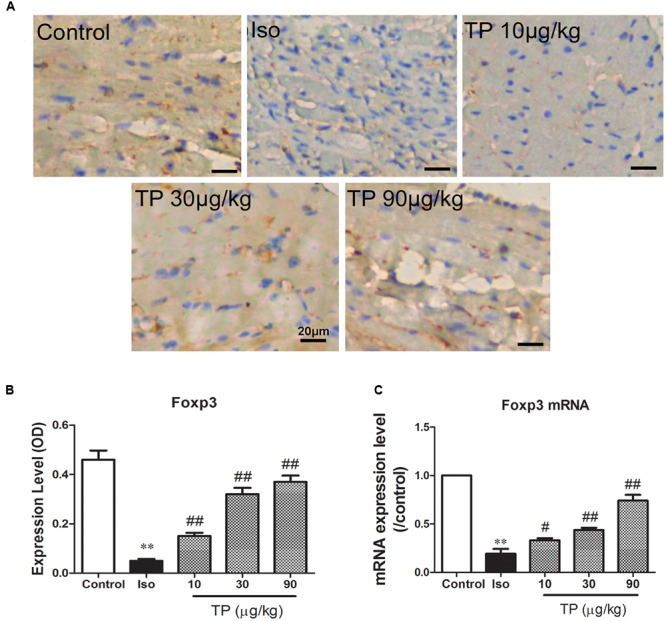
**Myocardial expression of transcription factor Foxp3.** Cardiac hypertrophy was induced by isoproterenol (5 mg/kg, s.c., for 14 days) in mice (*n* = 8–10 in each group). The mice were treated with normal saline, Iso, and triptolide (TP, 10, 30, 90 μg/kg), respectively, for 14 days. **(A)** Expression of Foxp3 was determined using immunohistochemistry (bar = 20 μm); **(B)** histogram represented the expression level of Foxp3 (relative integral optical density, OD); **(C)** Foxp3 mRNA expression level, determined with Real-time PCR. The data are presented as mean ± SEM, ^∗∗^*p* < 0.01 compared with the control group;^#^*p* < 0.05, ^##^*p* < 0.01 compared with the Iso-treated group (one-way ANOVA).

As shown in **Figure 7**, myocardial expression of transcription factor Foxp3 decreased markedly in isoproterenol-induced cardiac hypertrophy mice compared to those in the control group (*p* < 0.01). TP treatment significantly elevated protein expression of Foxp3. The elevations were correlated significantly with TP dosage (*r* = 0.861, *p* < 0.01).

## Discussion

TP is a major active component of *Tripterygium wilfordii* Hook F, which has been used for the treatment of autoimmune diseases for 1000s of years due to its anti-inflammatory and immunosuppressive effects (Liu, 2011). In recent years, studies have suggested that TP could cause a decrease in the GATA-4 DNA binding activity of nuclear factor of activated T cells (NFAT) and may effectively downregulate the activity of nuclear factor (NF)-κB (Qiu and Kao, 2003; Liu et al., 2014). NFAT and NF-κB are involved not only in regulating cardiac hypertrophy and ventricular remodeling independently, but also in promoting them synergistically through a direct interaction that integrates signal pathways leading to cardiac hypertrophy (Liu et al., 2012, 2014). In the present study, we found that TP could effectively alleviate myocardial damage, reduce the left ventricle index and inhibit the occurrence of myocardial hypertrophy in a dose-dependent manner.

Inflammation and immunity play an important role in the development of cardiac hypertrophy. It has become clear recently that immune imbalance including a decline in immune cells and factors, along with an obvious increase in immune cytokines, participates in the development of cardiac hypertrophy (Barhoumi et al., 2011). Many studies have demonstrated that immunoregulation and inflammation control could improve patients’ myocardial contractility, cardiac function and quality of life through correcting the imbalance of the lymphocyte subpopulation and inhibiting the myocardium damage induced by pro-inflammatory factors (Wallace et al., 2005; Rassi et al., 2009; Straburzyńska-Migaj, 2009; Wei, 2011). With the excellent effects of anti-inflammation and immunosuppression, TP can improve ventricular function significantly by downregulating NF-κB signaling (Wen et al., 2013).

The Fox transcription factor family is characterized by forkhead helix domain, which have various members that perform different functions. As a main member of this family, Foxp3 has been shown to execute function in regulating the inhibition of Treg cells and reducing the immune response (Stoop et al., 2007). Moreover, in addition to Treg cells, Foxp3 is found to be expressed in various other cells, such as many different types of cancer cells (Karanikas et al., 2008; Ladoire et al., 2011) and epithelial cells from mammary glands, lung bronchia and prostate glands (Chen et al., 2008). Foxp3 can associate physically with Rel transcription factors, such as NFAT and NF-κB, and block the endogenous expression of their target genes (Bettelli et al., 2005). However, heretofore, it was unclear whether Foxp3 was expressed in cardiomyocytes and whether Foxp3 was able to suppress the development of cardiac hypertrophy. In the present research, using colocalized immunofluorescence, we found that Foxp3 was expressed in cardiomyocytes, and its expression was downregulated by AngII stimulation. TP treatment elevated Foxp3 expression and its translocation from cytoplasm to nuclei. Immunohistochemistry and quantitative PCR showed that Foxp3 and its mRNA were expressed in normal myocardium, and myocardial expression of them was decreased markedly in cardiac hypertrophy mice. TP treatment significantly elevated mRNA and protein expression of Foxp3. By this mean, up-regulation of Foxp3 could directly inhibit NFAT activity in myocardial cells, which may attenuate the development of myocardial hypertrophy (Liu et al., 2012). Additionally, the anti-inflammatory and immunosuppressive effects of TP could also be involved in inhibiting the process of cardiac hypertrophy.

Our study still has certain limitations. Although the expression of Foxp3 were determined with colocalization immunofluorescence in cardiomyocytes and immunohistochemistry in myocardial tissue, more researches need to be performed to confirm the role and molecular mechanism of myocardial Foxp3 in the development of cardiac hypertrophy using the knockout animals and genetic inhibition technique. In addition, we observed the cross-sectional area of cardiomyocytes from histological sections by using FITC-labeled lectin staining. Lectin is a type of carbohydrate-binding protein, which may bind not only soluble extracellular and intercellular glycoprotein, but also the carbohydrate moity of glycolipid. This staining method is not as sensitive and accurate as vinculin immuohistochemistry for the measurement of cell area (Santos-Gallego et al., 2016). Vinculin is a membrane-cytoskeletal protein in focal adhesion plaques, which is more speciafically localized to the ends of actin filaments (Hirsch et al., 1996).

## Conclusion

Triptolide can effectively alleviate myocardial hypertrophy by inhibiting left ventricular remodeling, elevating the myocardial expression of Foxp3, and correcting the imbalance of expression of α-MHC and β-MHC. Transcription factor Foxp3 and inflammatory factors are involved in the process of cardiac hypertrophy and pathological remodeling, which provide a new approach for the prevention and treatment of cardiac hypertrophy.

## Author Contributions

H-GZ, X-HC, and YL designed and performed the experiments, drafted and revised the manuscript, and prepared the final version of the manuscript. Y-YD, J-ML, F-JG, YL, Y-FT, X-CP, X-LL, and WY performed the experiments and analyzed and interpreted the data. All authors read and approved the version submitted for publication.

## Conflict of Interest Statement

The authors declare that the research was conducted in the absence of any commercial or financial relationships that could be construed as a potential conflict of interest.

## Footnotes

1 https://www.aaalac.org/accreditation/RefResources/RR_HumaneEndpoints.pdf

2 http://rsbweb.nih.gov/ij/
